# Sustainable Green Extraction of Carotenoid Pigments: Innovative Technologies and Bio-Based Solvents

**DOI:** 10.3390/antiox13020239

**Published:** 2024-02-15

**Authors:** Ángeles Morón-Ortiz, Paula Mapelli-Brahm, Antonio J. Meléndez-Martínez

**Affiliations:** Food Colour and Quality Laboratory, Facultad de Farmacia, Universidad de Sevilla, 41012 Sevilla, Spain; amortiz@us.es (Á.M.-O.); ajmelendez@us.es (A.J.M.-M.)

**Keywords:** 2-methyltetrahydrofuran, ethyl lactate, ionic liquids, microwaves, natural deep eutectic solvents, pressurized liquid extraction, pulsed electric fields, subcritical fluid extraction, supercritical fluid extraction, ultrasounds

## Abstract

Carotenoids are ubiquitous and versatile isoprenoid compounds. The intake of foods rich in these pigments is often associated with health benefits, attributable to the provitamin A activity of some of them and different mechanisms. The importance of carotenoids and their derivatives for the production of foods and health-promotion through the diet is beyond doubt. In the new circular economy paradigm, the recovery of carotenoids in the biorefinery process is highly desirable, for which greener processes and solvents are being advocated for, considering the many studies being conducted at the laboratory scale. This review summarizes information on different extraction technologies (ultrasound, microwaves, pulsed electric fields, pressurized liquid extraction, sub- and supercritical fluid extraction, and enzyme-assisted extraction) and green solvents (ethyl lactate, 2-methyltetrahydrofuran, natural deep eutectic solvents, and ionic liquids), which are potential substitutes for more toxic and less environmentally friendly solvents. Additionally, it discusses the results of the latest studies on the sustainable green extraction of carotenoids. The conclusions drawn from the review indicate that while laboratory results are often promising, the scalability to real industrial scenarios poses a significant challenge. Furthermore, incorporating life cycle assessment analyses is crucial for a comprehensive evaluation of the sustainability of innovative extraction processes compared to industry-standard methods.

## 1. Introduction

Carotenoids are versatile compounds naturally found in food sources, including plant-derived (e.g., broccoli, tomato, pepper, carrot, or spinach) or animal-derived (e.g., egg yolk, salmon flesh, or mussels) foods. These compounds can also be found in other non-edible plants and animals, as well as in other matrices such as algae, some bacteria, and some fungi. With few exceptions, carotenoids are natural pigments, many of which are involved in many important biological actions [1]. They play roles in photoprotection, light absorbance, redox reactions, plant resilience to stress, seed dispersal, germination, and pollination, among others [2]. The diverse range of actions exhibited by carotenoids and their derivatives positions them as crucial compounds in food production, consequently playing a pivotal role in ensuring food security [1]. These bioactive compounds do not only stand out as natural pigments with many natural functions, but also act as health-promoting compounds through different mechanisms (light absorption, modulation of gene expression, interaction with reactive oxygen species, etc.), resulting in different effects, including antioxidants and anti-inflammatory effects. Certain carotenoids serve as precursors of vitamin A, considered the vitamin with the widest spectrum of actions. They are therefore key to combatting vitamin A deficiency, a major nutritional problem in the world [3]. In relation to these actions, substantial evidence indicates that these compounds contribute to providing various health benefits for humans, including immunity enhancements and potential reductions for the risk of developing several types of cancer, cardiovascular diseases, and bone, skin, and ocular disorders, among others [4,5]. 

As a consequence of the benefits associated with these compounds, they are of great interest for the production of functional foods nutraceuticals, nutricosmetics, supplements, botanicals, and novel foods [1,4]. To make these products, carotenoids are often extracted from sources where they are at high concentration. The selection of an appropriate solvent depends on the complexity of the matrix and the polarity of each carotenoid, among other factors. Conventionally, carotenoids are extracted using solvents such as hexane, acetone, tetrahydrofuran, petroleum ether, diethyl ether, chloroform, ethyl acetate, and ethanol; however, some of these solvents are considered toxic [6]. Given the increasing demand for carotenoids on an industrial scale, it is important to ensure economic viability and environmental sustainability in this process [6]. Thus, the use of green solvents (2-methyloxolane, ethyl lactate, and ionic liquids, among others) and green techniques that help reduce the consumption of energy and other resources (such as ultrasound-assisted extraction (UAE), microwave-assisted extraction (MAE), or pressurized liquid extraction (PLE)) has increased in recent years. Addressing the sustainability of the extraction process aligns with broader considerations in food science, health, and eco-friendly practices.

## 2. Methodology

The articles included in this review were retrieved from Scopus. The starting search strategy was as follows: Title: “Carotenoid” AND “each of the technologies/type of solvents” separated with “or”. In some cases, other references included in the works retrieved were also read and commented in the review. In order to keep the work to a reasonable length, when more than 10 references were found for a particular technology or type of solvent, some studies were left out. In such cases, it was procured to include matrices of special interest, usually for their dietary importance or innovative nature.

## 3. Green Chemistry and Green Extractions: The Concepts

Green chemistry is considered the development, design, and implementation of chemical products and processes to decrease or eliminate the usage and generation of hazardous substances [7]. Green chemistry has established guidelines for modern chemical processes that prioritize sustainability and environmental friendliness. The principles of green chemistry include minimizing waste, using renewable resources, preventing pollution, and designing chemicals and materials that are non-toxic, among others [8]. 

On the other hand, green extraction involves identifying and creating extraction methods that minimize energy usage, enable the utilization of renewable natural products and alternative solvents, and guarantee the extraction of high-quality and safe products. The principles of green extraction involve innovation through the selection of varieties and the use of renewable plant resources, which emphasizes the use of selecting plant varieties that are abundant and sustainable [9]. Green extraction promotes the use of alternative solvents, such as water or agro-solvents, and other bio-based solvents as alternatives to toxic and hazardous solvents, such as chlorinated solvents or benzene [10]. In addition, the reduction of energy consumption during the extraction process through energy recovery and the use of innovative technologies, such as microwave-assisted extraction (MAE), ultrasonic-assisted extraction (UAE), and supercritical fluid extraction (SFE), among others, is also an important goal. Moreover, the principles also comprise the production of co-products as an alternative to waste, the reduction of the number of experiments, and the obtaining of non-denatured and biodegradable extracts without contaminants [7,9]. Integrating the green extraction practices of natural products with the principles of green chemistry can lead to the development of a “Green Chemistry of Natural Products”, which could promote the use of natural resources and reduce the negative impact of chemical processes on the environment and human health to address global challenges [9]. Overall, the concepts of green chemistry and green extraction are aimed at promoting sustainable and environmentally friendly practices in chemistry and chemical engineering, with applications in other fields and industries, such as agri-food, pharma, and cosmetics, among others. 

## 4. Technologies Amenable to Green Extractions

**Figure 1:**
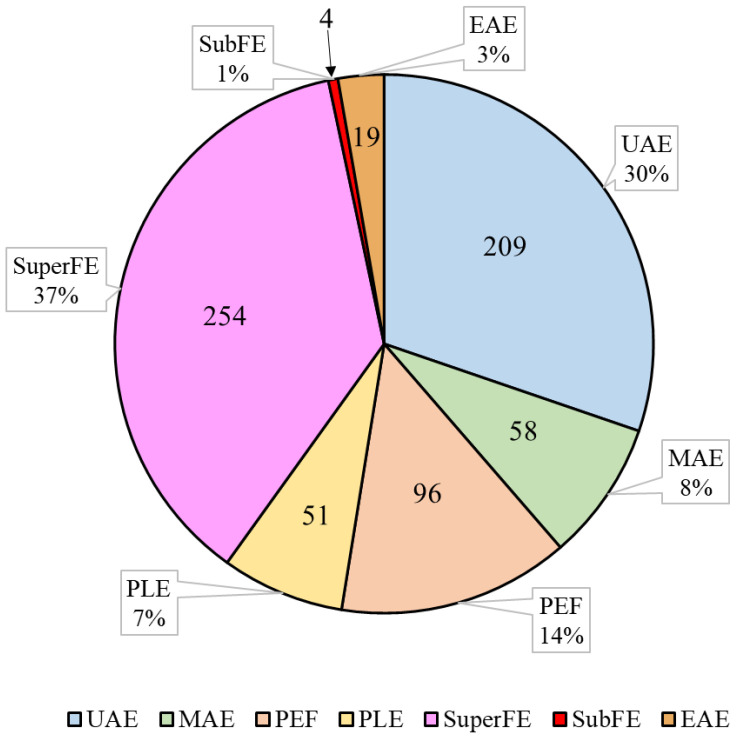
Number (inside the chart) and percentage (outside the chart) of published research articles related to carotenoid extraction using the respective methodologies. UAE: ultrasound-assisted extraction; MAE: microwave-assisted extraction; PEF: pulsed electric field; PLE: pressurized liquid extraction; SuperFE: supercritical fluid extraction; SubFE: subcritical fluid extraction; EAE: enzyme-assisted extraction. Source for article search: Scopus.

Green extraction technologies have been employed for carotenoid recovery from diverse matrices, since they enhance carotenoid extraction through various extraction mechanisms (Table 1). Among these methodologies, ultrasound-assisted extraction and supercritical fluid extraction have garnered significant attention in scientific investigations, being extensively explored compared to other alternative sustainable techniques (i.e., microwave-assisted extraction, pulsed electric field, pressurized liquid extraction, subcritical fluid extraction, and enzyme-assisted extraction), as depicted in Figure 1. The main results of the studies on carotenoid recovery with green extraction methodologies discussed in this review are summarized in Table 2.

### 4.1. Ultrasounds

Ultrasound is a type of sound wave with a frequency above the audible range of human hearing, typically above 20 kHz. Ultrasound can be differentiated into low- or high-frequency. High-frequency ultrasound typically operates at frequencies higher than 1 MHz and is used for medical practices, such as imaging, diagnostic, and therapeutic applications. On the other hand, low-frequency ultrasound typically operates at frequencies below 100 kHz and is often used for applications such as cleaning, emulsification, and deagglomeration. Low-frequency ultrasound causes the formation of the phenomenon called “cavitation”, which results in the rapid formation and implosion of small bubbles in a liquid, which can disrupt cellular structures and facilitate the release of compounds from the sample, enhancing the extraction efficiency of compounds from solid matrices. Thus, it is used to extract compounds from different matrices, as it can penetrate deep into the matrix and release intracellular contents. Compared to conventional extraction techniques (maceration, Soxhlet, etc.), ultrasound-assisted extraction (UAE) improves the extraction efficiency, reduces the extraction time and energy, and increases the extracted yield [11,48]. The bath and probe ultrasound instruments are the most common for the food industry. The bath method involves submerging the sample in a liquid through which the ultrasound is applied, while the probe ultrasound method involves using a handheld probe immersed in the sample. Both methods can be effective in generating cavitation and disrupting cell walls to release bioactive compounds, but the choice of an appropriate solvent and other parameters should be optimized to maximize extraction efficiency while minimizing potential undesirable effects [49].

Several studies have compared the extraction of carotenoids via UAE and conventional extraction methods, regarding time, solvent, and extraction yield [18,19,20]. For example, the content of trans-lutein obtained from pumpkin peel via an optimized UAE (45 kHz, 210 W, 30 min, ethanol–petroleum ether mixture (2:1, *v*/*v*) as extraction solvent, and a solvent-to-solid ratio of 31 mL/g) was 1.15 folds higher than that obtained via an optimized conventional solvent extraction (40 °C, 90 min under reflux condition, ethanol–petroleum ether mixture (2:1, *v*/*v*) as extraction solvent, and a solvent-to-solid of 20 mL/0.5 g). Thus, UAE enhances carotenoid extraction yield from pumpkin peel in less time and with less use of solvent [21]. 

In a recent study, the effectiveness of an optimized UAE (40 kHz, 80 W, 19 min, and a mixture of 44% acetone and 56% methanol as extraction solvent) was compared to that of an optimized conventional extraction (maceration under mechanical agitation, 23 min, and a mixture of 38% acetone, 30% ethanol, and 32% petroleum ether as extraction solvent) for the extraction of carotenoids from cashew apples. The results indicated that the UAE yielded a significantly higher carotenoid content. Specifically, carotenoid extraction from yellow, red, and yellow/red mixture (1:1; *w*:*w*) cashew apples using the optimized UAE resulted in 1.4, 1.3, and 1.2 times increases in yield, respectively, compared to the optimized conventional extraction (maceration under mechanical agitation) [18]. In another study performed on orange peels, 68 hydrophobic and hydrophilic deep eutectic solvents were screened to select the best solvent for the extraction of carotenoids by considering the efficiency, stability, and physicochemical properties. The mixture menthol/camphor in a molar ratio of 1:1 was selected as the best extraction solvent for the UAE. With this solvent, the use of an optimized UAE (120 W, 20 mL/g of solvent-to-solid, and 20 min) resulted in an approximately two-fold increase in yield compared to that of an optimized conventional extraction (maceration under mechanical agitation (150 rpm), room temperature, 10 mL/g of solvent-to-solid ratio, and 30 min) [19]. The optimization of a UAE methodology for the recovery of carotenoids from cantaloupe rind using response surface methodology (RSM) has been described. The effects of different factors, such as solvent mixture, extraction time, amplitude, and solvent-to-solid ratio, on the extraction yield were evaluated. A Central Composite Design (CCD) was considered to optimize the extraction conditions. Four independent variables (hexane percentage in the solvent mixture (hexane/acetone (1/1, *v*/*v*), hexane/ethanol (1/1, *v*/*v*), and hexane/acetone/ethanol (2/1/1, *v*/*v*/*v*)), extraction time (10, 20, 30, and 40 min), amplitude (20, 40, 60, 80, and 100%), and solvent-to-solid ratio (30, 40, 50, and 60 mL/g)) were tested at three levels. A regression analysis indicated that the hexane percentage and ultrasound amplitude did not significantly influence the extraction yield, but their interaction did. The solvent-to-solid ratio had a significant influence. The best solvent mixture for carotenoid extraction was hexane/acetone, so different proportions were tested: 50/50; 70/30; and 90/10 (%, *v*/*v*). The optimized parameters were set as follows: a hexane/acetone proportion of 80:20 (*v*/*v*), time of 10 min, amplitude of 100%, and solvent-to-solid ratio of 55 mL/g [22]. 

The optimization of the UAE of carotenoids in buriti pulp and carrot pomace has also been studied [23,24]. For instance, the optimized conditions for the carotenoid extraction from buriti pulp were 30 min, at a 40 kHz ultrasound frequency, power of 80 W, and a mixture of acetone/ethanol (75:25) as the extraction solvent with a solid-to-solvent ratio of 8:10, leading to a total carotenoid extraction of 1026 µg/g [23]. In the case of freeze-dried carrot pomace, the UAE of carotenoids was optimized. In preliminary studies, five solvents (methanol, acetone, ethanol, acetonitrile, and *n*-hexane) and three sample-to-solvent ratios (1:30, 1:50, and 1:70 g/mL) were evaluated, selecting ethanol at a solid-to-solvent ratio of 1 g/50 mL as the best condition for the extraction. Then, UAE (at 70% and 20 kHz) was optimized using the CCD, evaluating different ethanol concentrations (13–97%), temperatures (10–60 °C), and extraction times (3–37 min). The optimized parameters were 51% of ethanol concentration, 32 °C, and 17 min. The extracted carotenoid yield with these optimized conditions was 31.82 µg/g [24]. 

Studies about UAE with emerging environmentally friendly solvents are also being carried out. The extraction of fucoxanthin from *Sargassum fusiforme* via UAE with different solvents (ethanol/acetone (3:1, *v*/*v*), ethyl lactate, limonene, soybean oil, and sunflower oil) was evaluated. It was observed that ethyl lactate performed similarly to the traditional organic solvent evaluated (ethanol/acetone (3:1, *v*/*v*). Then, RSM was applied to select the best conditions of time, amplitude, and temperature for the UAE at 20 kHz, using 40 millilitre of ethyl lactate per gram of sample. The best conditions for the fucoxanthin extraction were 27 min (extraction time), 75 °C (extraction temperature), and 53% (amplitude) [20]. The effect of milling (30 Hz, 5 min) and the performance of common (ethanol, methanol, dimethyl sulfoxide) vs. emerging green solvents (ethyl lactate, 2-methyl oxolane) in the UAE of wild-type and phytoene-rich *Chlorella sorokiniana* microalgae have recently been assessed. Three formulations (fresh, freeze-dried, and encapsulated in alginate) of wild-type and phytoene-rich *C. sorokiniana* were used. Significant differences among the extraction efficiencies of some solvents were reported, 2-methyl oxolane being one of the most efficient solvents for the extraction of carotenoids. The milling pretreatment significantly improved carotenoid extraction in freeze-dried and encapsulated matrices, but there was no effect on the fresh matrices [25]. 

All in all, increasing evidence is accumulating on that the UAE of carotenoids from different food matrices can be a sustainable technique suitable for replacing conventional extractions in which a higher energy consumption, longer extraction time, and larger volume of extraction solvent are generally required. 

### 4.2. Microwaves

Microwave-assisted extraction (MAE) is a technique that enhances the extraction process by utilizing microwave heating. Microwave heating occurs through heat dissipation through irradiation; in contrast, in conventional heating methods, heat is transferred from the heating device to the sample via conduction or convection. As a result, microwave heating is often able to achieve higher temperatures faster than conventional methods [12]. The amount of energy dissipated during MAE depends on the electric field strength, with frequencies in the range of 300 MHz–300 GHz, and the dielectric properties of the sample.

The microwave radiation penetrates the sample, and it converts the electromagnetic energy into thermal energy through two mechanisms, ionic conduction and dipole rotation. The agitation of the sample molecules that MAE generates leads to an increase in temperature. Although MAE has been shown to be a promising technique for improving the efficiency and time of the extraction process compared to conventional extraction methods, there are some limitations to the extraction of heat-sensitive compounds [50]. Therefore, the choice of solvent is critical to controlling the efficiency and temperature of the MAE process. Solvents with high dielectric constants, such as water, ethanol, and methanol, may overheat when using long irradiation times, potentially harming thermolabile compounds [51]. 

There are different MAE methods, including closed-system extraction and open-system extraction. Both systems have their advantages and disadvantages. The closed system (multimode) uses high pressures and temperatures, allowing a fast and efficient extraction with a lower solvent consumption. However, this system is not capable of processing a large number of samples and may result in losses of volatile compounds. The open system (mono-mode) operates under atmospheric conditions and is considered to be more suitable for extracting thermolabile compounds, such as carotenoids. This system has a higher sample throughput, as more samples can be processed compared to the closed system, and more solvent can be added at any time during the process [12].

MAE can reduce the extraction time and energy consumption and increase the yield of target compounds compared to traditional extraction methods [25,51]. For instance, the extractions of lutein from *C. sorokiniana* with ethanol as extraction solvent after an alkali-assisted microwave extraction pretreatment and after a conventional alkali digestion pretreatment have been compared. The optimized MAE method (37 mg microalga/mL aqueous KOH, 8 M KOH, 60 °C, and 2 min) resulted in an extraction yield 3.3 folds higher than conventional extraction (20 mg microalga/mL aqueous KOH, 10 M KOH, maceration in a water bath at 60 °C for 30 min) [26]. The MAE conditions have also been optimized for several bioactive compounds from different matrices, such as carrot waste using flaxseed oil as an extraction solvent, which resulted in a carotenoid recovery of 77.48% under optimal conditions of 165 W of microwave power, 9.39 min of extraction time, and an oil-to-waste ratio of 8.06:1 (g/g). The regression coefficient values indicated that the highest influence on the recovery of carotenoids was the extraction time, followed by the microwave power [27]. Additionally, MAE conditions for carotenoid extraction from *Aristeus antennatus* shrimp were optimized, resulting in the successful recovery of 13.3 mg of carotenoids per 100 g of the dry sample under the following conditions: a mixture of hexane, acetone, and ethanol (2:1:1, *v*/*v*/*v*) as the extraction solvent, temperature of 50–52 °C, microwave power of 30 W, extraction time of 7 min, material-to-solvent ratio of 1:20 (*w*/*v*), and no ramping time [28]. 

### 4.3. Pulsed Electric Fields

Pulsed electric field (PEF) is a method that uses low energy to open the cell membranes of food, allowing for a better transfer of substances in and out of the cells. PEF has been studied for its ability to improve food quality without damaging its compounds; thus, there are many potential uses of PEF in the food industry for improving food processing [52]. 

PEF-assisted extraction can be performed through two main methods, batch and continuous flow systems. Batch systems involve exposing the sample to high-voltage pulses for a set time, while continuous flow systems subject the sample to high-voltage pulses as it flows through a chamber through a series of electrodes. Both methods offer advantages over traditional extraction methods by allowing a higher throughput, reduced processing time, and higher product quality. In addition, PEF is considered an environmentally friendly method, as it reduces the use of solvents and energy consumption compared to other traditional extraction methods [13]. 

The use of PEF as an extraction method has been shown to be effective in increasing the release of a wide range of intracellular compounds, such as lipids, proteins, carbohydrates, and pigments from bacteria, yeast, and microalgae [29].

In a study performed by Martínez et al. (2019), the extraction of carotenoids from *Haematococcus pluvialis* with ethanol after a PEF pretreatment (1 kV/cm, 10 pulses, 50 kJ/kg) in its own cultivation medium followed by incubation in a growth medium for 1, 6, or 12 h was investigated. PEF pretreatment followed by incubation for 6 h increased the yield 2.4-fold compared to untreated samples (also extracted with ethanol). This PEF pretreatment was also compared with other four pretreatments: bead beating (5 or 10 cycles of 60 s at 4800 rpm, with water), freeze-thawing (freezing in liquid nitrogen, melting on ice, 5 cycles), thermal treatment (70 °C, 1 h), and ultrasound (10 cycles, 10 s, 80% amplitude, 450 W, with ethanol). PEF pretreatment resulted in the best carotenoid extraction [30]. In another study, the extraction of carotenoids from tomato peel treated with PEF (electric field of 5 kV/cm, a solid-to-solvent ratio of 5 g/100 mL, and treatment time of 90 µs) using hexane/acetone/ethanol (50:25:25) or hexane/ethanol (50:50) as extraction solvents resulted in approximately 1.6 and 1.7 higher carotenoid recoveries, respectively, compared to that of the conventional solvent extraction method (maceration in a water bath at 25 °C and at 120 rpm, using the same solvents and with the same solid-to-solvent ratios) [31]. In another research, the extraction of carotenoids from *C. vulgaris* with ethanol (maceration, 20 min, room temperature, sample-to-solvent ratio of 1:10 (*v*/*v*)) was compared in non-pretreated samples and PEF-pretreated samples (20 kV/cm and 75 µs) just after the PEF treatment or after pre-incubation of cells at 20 °C in the treatment medium for 1 h after applying the PEF treatment. The extraction yield was 42% higher when the extraction was conducted after 1 h of pre-incubation compared to the extraction just after the PEF treatment. The PEF-pretreatment of samples after 1 h of incubation significantly increased the extraction yield of the carotenoids by 124% compared to the untreated samples [32]. 

The use of PEF (3 kV/cm, 100 kJ/kg, 74 pulses, solid-to-solvent ratio 1:10 (g/mL)) for the extraction of carotenoids from side streams of red shrimp (*Aristeus antennatus*) and camarote (*Melicertus kerathurus*) has recently been reported. Ethanol and dimethyl sulfoxide (DMSO) were compared, the latter leading to higher recoveries. PEF with DMSO and ethanol significantly increased the recovery of astaxanthin in *M. kerathurus* and *A. antennatus* compared to the control (1:10 (*w*/*v*), stirred at 400 rpm, 30 min, using both DMSO and ethanol) [33].

### 4.4. Pressurized Liquid Extraction 

Pressurized liquid extraction (PLE), also known as accelerated solvent extraction (ASE), uses liquid solvents at high pressures (typically 3.5 to 20 MPa) and temperatures (between 50 and 200 °C) to extract compounds from solid or semi-solid matrices [53].

The PLE process consists of three steps: static extraction, dynamic extraction, and separation and detection. In the first step, a sealed container (containing supercritical CO_2_ and a modifier or co-solvent, which have been pumped before) is heated to a set temperature, leading to a release of the compounds. The dynamic extraction process involves the flow of solvent through the extraction vessel in a direction opposite to that of the static extraction. This transfers the extracted compounds to an analytical column for separation and detection. To aid in the ionization of the extracted compounds, an organic solvent is added to the mobile phase along with a modifier, as carbon dioxide alone is not sufficient for this purpose [54].

In a recent study, PLE (preheating: 1 min, heating: 5 min, flush volume: 60%, nitrogen purge: 60 s, pressure: 103.4 bars, 0.5 g sample/20 mL solvent, 40 °C, 15 min extraction time) was successfully used for the recovery of carotenoids from dried microalgae (*Chlorella vulgaris*, *Phaeodactylum tricornutum*, and *Spirulina maxima*) using DMSO as extraction solvent. A significant increase in carotenoid extraction compared to conventional extraction (maceration with the same sample-to-solvent ratio, temperature, extraction solvent, and extraction time) was observed, being approximately 3, 3.3, and 5.9 times higher in *C. vulgaris*, *P. tricornutum*, and *S. maxima*, respectively [34]. In another study, similar findings were found in freeze-dried Spirulina extracted with DMSO (100%) via PLE and conventional extraction. The carotenoid content in PLE-treated samples (preheating 1 min, heating 5 min, flush volume 60%, nitrogen purge 60 s, pressure 103.4 bars, 0.5 g sample/20 mL solvent, 40 °C, and 15 min extraction time) was significantly higher (approximately 5.6-fold) compared to that in samples extracted by a conventional extraction method (stirring maceration, 0.5 g sample/20 mL solvent, 40 °C, 15 min) [35]. In addition, fucoxanthin from dry and wet *Phaeodactylum tricornutum* has also been satisfactorily extracted via PLE (pressure: 103.4 bars, extraction time: 20 min, rinse volume: 60%, nitrogen purge: 300 s, extraction temperature: 100 °C, biomass/solvent/water content set as 1/14/0 and 1/12/3 (g/mL/mL) for dry and wet biomasses, respectively) using ethanol, ethyl acetate, and *n*-hexane as solvents. The findings showed similar fucoxanthin yields between samples extracted with ethanol and ethyl acetate in dry biomass; nevertheless, a significantly higher fucoxanthin extraction was observed in wet biomass using ethanol than ethyl acetate. n-Hexane was the least efficient solvent for fucoxanthin extraction in *P. tricornutum* among the three solvents evaluated [36].

The use of ASE (1 min preheating, 5 min heating, 60% flush volume, pressure of 10 MPa for 15 min, 60 s for nitrogen purge, 50 °C temperature extraction) for the extraction of carotenoids from red shrimp (*A. antennatus*) and camarote (*M. kerathurus*) side streams was also studied. Ethanol and DMSO were compared, the latter proving a more efficient extraction. ASE resulted in approximately three-fold and two-fold increased in the amountd of astaxanthin extracted in *A. antennatus* and *M. kerathurus*, respectively, compared to the control extraction (maceration, 1:10 (*w*/*v*), stirred at 400 rpm, 30 min, using both DMSO and ethanol). In this study, the combination of PEF and ASE were also assessed, using DMSO as the extraction solvent, which resulted in the highest astaxanthin extractions in both *M. kerathurus* and *A. antennatus*, recovering 213.1 and 585.9 µg/g dw, respectively [33].

Thus, PLE can be considered a potentially amenable technique as a sustainable method for the extraction of carotenoids, as it could improve the extraction efficiency and reduce the extraction time and energy usage compared to traditional methods.

### 4.5. Supercritical Fluid Extraction

Supercritical fluid extraction (SFE) is based on the use of a substance, known as supercritical fluid, which is maintained above its critical temperature and pressure. This unique state allows the supercritical fluid to exhibit both gas and liquid properties. By subjecting the sample to high pressures and temperatures in the extraction chamber, the supercritical fluid can enhance the solubility of the targeted compounds due to those dual properties as a liquid and a gas [15]. The most widely used supercritical fluid is carbon dioxide, although others such as hydrocarbons, aromatic compounds, and alcohols have also been considered [55]. Supercritical fluids are considered ideal extraction solvents due to their unique properties, such as high diffusivity, low viscosity, and low surface tension. These properties allow them to penetrate the sample matrix and dissolve target compounds more efficiently than other solvents. In addition, they have a high density, which improves their solubility and extraction efficiency [55], as well as reduces the energy expenditure compared to other conventional extraction methods [48]. 

SFE can be applied in three different types of tank reactors: batch, semi-continuous, and continuous. Batch tank reactors have a large volume and can last several hours or a day to complete an extraction. Semi-batch tank reactors operate continuously for only part of the process, while continuous flow reactors continuously pump feedstock into the reactor. Continuous flow reactors provide precise temperature and residence time control and enable the use of large volumes in the tank reactor in a short time [56].

In a study performed by Nunes et al. (2021) on brown crab processing waste, the astaxanthin concentration obtained via SFE (500 bars, 40 °C, 13% ethanol content in supercritical fluid mixture, 90 min extraction time) was approximately 22.5 times higher than that obtained through conventional solvent extraction (1 g/mL solid-to-liquid ratio, stirring maceration at 40 °C for 30 min) [37]. The effects of the SFE (10% (*w*/*w*) of ethanol, 250 bars, 60 °C, flow rate of 40 g/min, and total solvent consumption of 100 kg CO_2_/ kg biomass) on the extraction of carotenoids from *C. vulgaris* were also evaluated. According to this study, the use of this method resulted in approximately 1.8- and 1.4-fold increases in the total amounts of extracted carotenoids when compared to that of a conventional solid–liquid extraction (aqueous ethanol 90% (*v*/*v*), 24 h) and a MAE method (60 °C, 300 W, 14 min, and 22 mL/g (solvent/biomass)), respectively [38]. The SFE of carotenoids has been also optimized in several matrices, such as pink grapefruit (325 bar, 64 °C, and 143 min, rice bran oil as co-solvent, CO_2_ and rice bran oil flow rate set at 35 g/min, and 3% of CO_2_ (*w*/*w*)) or carrot peel (349 bar, 80 min, 59 °C, 15.5% ethanol as co-solvent, CO_2_ flow rate of 15 g/min) [39,40]. 

### 4.6. Subcritical Fluid Extraction

Subcritical fluid extraction is a method of extracting compounds similar to SFE but works at lower pressures and temperatures. The solvents used in subcritical fluid extraction, such as propane, dimethyl ether, butane, or even water, are in a liquefied state, but below their critical temperature and pressure [57,58]. Studies have shown that CO_2_, 1,1,1,2-tetrafluoromethane, and dimethyl ether have the potential to extract carotenoids from algae using subcritical fluid extraction [57]. For instance, the optimized application of subcritical fluid extraction of carotenoids from *Laminaria japonica* with ethanol-modified subcritical 1,1,1,2-tetrafluoromethane (R134a) as the solvent (51 °C, 17 MPa, and 4.73% of ethanol as co-solvent), resulted in a yield of carotenoid of 0.233 g/kg. However, the yield obtained was higher (0.336 g/kg) with the UAE method using an ultrasound bath (methanol as extraction solvent, solvent-to-solid ratio of 10 mL/0.5 g) [41]. The use of subcritical fluid extraction (200:1 solvent-to-solid ratio, 120 rpm, 20 MPa pressure, 35 °C, 60 min) has been demonstrated to be an effective method for extracting fucoxanthin from the microalgae *Phaeodactylum tricornutum* using methanol as an extraction solvent (0.69 mg fucoxanthin/g wet cell weight). In this research, it has been found that this method enabled most of the cells to be disrupted and intracellular components to be effectively released [42]. Another study has shown that the subcritical propane extraction (293.15 K; 2 MPa) of β-carotene from *Maximiliana maripa* resulted in a 1.1 times higher extraction yield compared to supercritical petroleum ether extraction [43]. Moreover, previous investigations conducted on Ambara, Majdool, and Sagai date fruits have demonstrated that subcritical extraction (250 extraction cycles, 29 °C, 6.8 MPa, 12 h, and ethanol as solvent) resulted in significantly higher total carotenoid content when compared to Soxhlet (70 °C, 16 h, and *n*-hexane as solvent) or SFE (52.5 °C, 27.50 MPa, and 5 mL CO_2_/min of flow rate) methods, being 1.8–2.1 times and 1.0–1.2 times higher, respectively [44].

### 4.7. Enzyme-Assisted Extraction 

Enzyme-assisted extraction is a method that uses enzymes to break down the cell walls and membranes of the sample matrix, which can enhance the release of compounds. The enzymes are typically added to the sample matrix and allowed to incubate, under specific conditions of temperature, pH, time, and enzyme concentration, to optimize enzymatic activity and facilitate the release of the compound of interest [59]. Enzyme-assisted extraction is a greener option and has potential commercial benefits, as it could increase yields and enhance product quality using milder processing conditions. Moreover, this extraction method requires less energy than other traditional extraction methods [60]. However, it has some limitations, such as the high cost of enzymes, the incomplete hydrolysis of plant cell walls, and the difficulty in scaling up to an industrial level [59].

In a study carried out by Zuorro et al. (2011), it was observed that the use of mixed food-grade enzyme preparations with cellulolytic and pectinolytic activities (30 °C, 3.18 h extraction time, 0.16 kg enzyme load/kg partially dried tomato skins) greatly enhanced the recovery of lycopene from tomato peel waste compared to conventional extraction (maceration at 30 °C for 2.5 h), both using hexane as an extraction solvent (60 dm^3^/kg of solvent-to-solid ratio). In particular, the mixture cellulolytic/pectinolytic (50:50 resulted in an 8- to 18-fold increase in extraction yield [45].

The extraction of carotenoids through enzyme-assisted extraction has been optimized in several matrices. The optimized parameters for the extraction of carotenoids from carrot juice using fructozym^®^ MA were an enzyme concentration of 0.3 mL/100 g, 24 h, 37 °C, and pH 7.4, which resulted in a carotenoid yield of 393.2 µg/mL [46]. In addition, the extraction of lycopene from industrial tomato waste using an enzymatic pretreatment followed by ethyl acetate extraction has also been optimized through RSM. The optimized parameters for the enzyme pretreatment were determined as celluclast/pectinex (1:1), an enzyme/substrate ratio of 0.2 mL/g, 5 h of enzymatic reaction time, 40 °C, and pH 4.5. The optimized parameters for the extraction with ethyl acetate were as follows: a solvent–substrate ratio of 5 mL/g, 1 h extraction in a shaker, and room temperature. This method results in a recovery of 9.16 mg lycopene/g tomato waste. On the other hand, lycopene could not be detected in the matrix extracted without the enzymatic pretreatment [47].

## 5. Extraction of Carotenoids with Bio-Based Solvents

The choice of solvent for carotenoid extraction is crucial to achieve an efficient extraction and high yields [6]. In light of the current environmental challenges, the selection of an appropriate extraction solvent should consider not only its effectiveness, but also its environmental impact. Prioritizing the environmental impacts of extraction solvents is crucial in mitigating the impact of extraction processes on the environment. 

Bio-based solvents are derived from renewable biomass sources, such as crops, forestry residues, and other plant-based materials. These types of solvents are gaining popularity as an alternative to other traditional solvents derived from petrochemicals, which are non-renewable and have negative environmental impacts [61]. 

**Figure 2:**
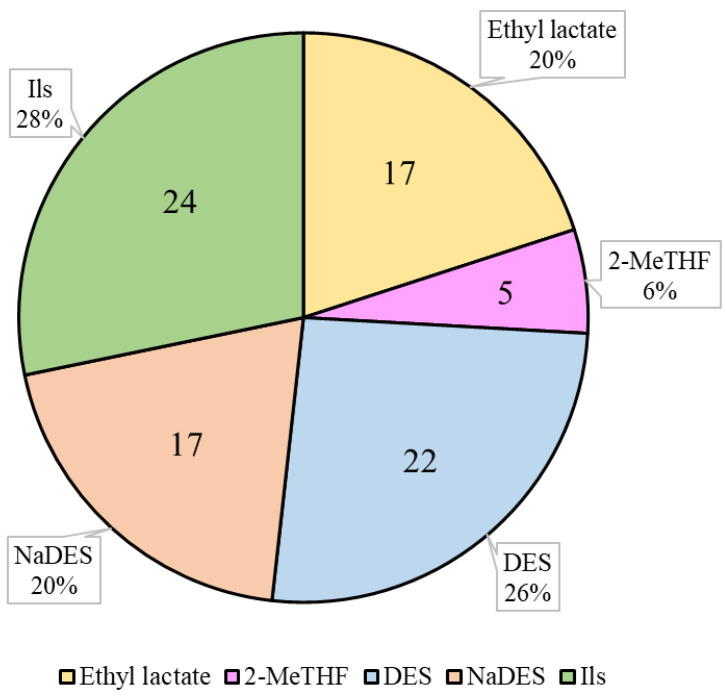
Number (inside the chart) and percentage (outside the chart) of published research articles related to carotenoid extraction using the respective solvent. 2-MeTHF: 2-Methyltetrahydrofuran; DES: deep eutectic solvent; NaDES: natural deep eutectic solvent; ILs: ionic liquids. Source for article search: Scopus.

The use of bio-based solvents has several advantages over other traditional solvents. Firstly, bio-based solvents are usually more sustainable and environmentally friendly, as they are derived from renewable resources. In addition, they generally have lower toxicity and are safer for human health. Finally, in some cases, they may have higher solubility and better compatibility with other chemicals. However, bio-based solvents also have some disadvantages, mainly related to the cost of production, which can be higher than that of other traditional solvents due to the higher cost of sourcing and processing renewable biomass [62,63]. The use of bio-based solvents for the extraction of carotenoids has been broadly studied and, overall, offers several advantages over petroleum-derived solvents in terms of environment [64]. The following bio-based solvents that have been used to extract carotenoids will be evaluated in this review: ethyl lactate, 2-methyltetrahydrofuran (2-MeTHF), natural deep eutectic solvents (NaDESs), deep eutectic solvents (DESs), and ionic liquids (ILs) (Figure 2). The main results of the studies on carotenoid recovery with bio-based solvents discussed in this review are summarized in Table 3.

### 5.1. Ethyl Lactate

Ethyl lactate is an organic compound that occurs naturally in small amounts in certain foods, such as poultry, fruits, and wine. It can be obtained by esterifying lactic acid and ethanol at high temperatures (80–120 °C) and low pressure and using a catalyst, such as sodium hydroxide. Ethyl lactate is separated from the resulting mixture via distillation. As the production has a low environmental impact and the lactic acid used can be obtained through the fermentation of sugars by certain microorganisms, such as lactic acid bacteria, ethyl lactate is considered a renewable alternative to petroleum-derived solvents [78,79]. Ethyl lactate is biodegradable, which means that it can be broken down by water or microorganisms in the environment. In addition, it is considered a non-toxic solvent [80]. Its physical and chemical properties make it desirable for carotenoid extraction (Table 4).

Ethyl lactate has been used for carotenoid extraction from different matrices such as dry tomato waste, crude palm olein (CPO), and *C. sorokiniana*, among others [25,65,66]. In particular, 17 research articles have evaluated the extraction of carotenoids with this solvent (Figure 2). This number represents 20% of the studies in which carotenoids are extracted with the bio-based solvents evaluated in this review (NaDESs, DESs, ethyl lactate, ILs, and MeTHF). Taking into account that NaDESs, DESs, and ILs represent a set of solvents, it is likely that ethyl lactate is the most researched bio-based solvent (among those evaluated in this review) for the extraction of carotenoids (Figure 2). 

It was observed that in a conventional carotenoid extraction (30 min, 70 °C) of dry tomato waste, the carotenoid yield obtained with ethyl lactate (243.00 mg/kg) was much higher than those obtained with acetone (51.90 mg/kg), ethyl acetate (46.21 mg/kg), hexane (34.45 mg/kg), and ethanol (17.57 mg/kg) [65]. In CPO, the extraction of carotenoids and tocols was optimized using a mixture of ethyl lactate and ethanol (3:2, *v*/*v*), setting the optimal parameters as 20 °C, 10 min of mixing (at 360 rpm), and 1 mL CPO/mL extraction solvent [66]. Finally, in encapsulated *C. sorokiniana*, ethyl lactate recovered a higher total carotenoid content compared to 2-MeTHF, ethanol, methanol, and DMSO, although significant differences were found only with ethanol and DMSO [25]. 

Szabo et al. (2022) found that in the lycopene extraction using an ultrasonic bath (10 min at 35 °C), the use of ethyl lactate as a solvent resulted in 1.4- and 2.5-fold increases in the extraction yield compared to hexane in fresh and freeze-dried tomatoes, respectively [67]. Furthermore, it has been suggested that ethyl lactate is a suitable solvent for extracting both *trans*- and *cis*-lycopene from lyophilized tomatoes using a temperature-controlled water bath (45 °C for 1 h) [68]. In another study, the total carotenoid content extracted from freeze-dried goji berries via UAE (40 °C, 30 min, 35 kHz) using ethyl lactate as the extraction solvent was found to be significantly higher than those extracted with ethanol (2.4–7.0 folds higher), acetone (2.6–8.7 folds higher), and sunflower oil (1.2–1.6 folds higher) [69]. These findings highlight the potential of ethyl lactate as a promising solvent for the extraction of carotenoids from different sources. 

### 5.2. 2-Methyltetahydrofuran

2-Methyltetrahydrofuran (2-MeTHF) has recently gained attention, as its synthesis pathway is produced from inedible renewable resources. It is known to degrade when exposed to air and sunlight, forming cellulose precursors such as xylose and glucose, although the mechanism of degradation is poorly understood [84]. 2-MeTHF has different uses in biolubricants, fuel additives, reagents, polymer precursors, foam materials, resins, environmentally friendly solvents, plasticizers, flavour and food additives, coatings, pharmaceuticals, cosmetics, surfactants, and intermediates for the manufacturing of herbicides and polycarbonates. Its diverse properties and broad applications make it a valuable compound for research and development in the chemical industry (Table 4) [85]. Its high boiling point (82 °C) can be advantageous for certain purposes, as it reduces the reaction time and enables reactions that require higher temperatures and its separation from water via distillation without adding drying agents. In addition, its low miscibility allows a clean workup. Furthermore, the production of 2-MeTHF from renewable sources has a low carbon footprint and has no known genotoxic or mutagenic effects [84,86]. This green solvent has been recently assessed by the European Food Safety Authority (EFSA) in terms of toxicity, concluding that a daily intake lower than 1 mg 2-MeTHF/kg body weight has no safety concerns [87]. 

2-MeTHF has recently emerged as a potential alternative to conventional organic solvents, such as *n*-hexane, for the extraction of various bioactive compounds, including carotenoids, from natural sources [70]. There are limited studies on the extraction of carotenoids with 2-MeTHF, representing 6% of the studies in which carotenoids are extracted with the bio-based solvents evaluated in this review (NaDESs, DESs, ethyl lactate, and ILs) (Figure 2). Kashyap et al. (2022) concluded that 2-MeTHF is a promising alternative to *n*-hexane and tetrahydrofuran for the extraction of lutein throughout maceration extraction (three washes: 1:10 (*w*/*v*) 3 h, 1:5 (*w*/*v*) 3 h; 1:5 (*w*/*v*) 24 h) from flowers of *Tagetes erecta* [71]. In a recent study, it was reported that the extraction of olive pomace using the Soxhlet extraction technique (4.5 h) with aqueous 2-MeTHF (95.5%) and dry 2-MeTHF (recovered via distillation), resulted in 11.2 folds and 12.5 folds higher carotenoid concentrations, respectively, compared to those obtained using *n*-hexane [72]. Yara-Varon et al. reported that a conventional solid–liquid extraction via maceration (65 °C, 1 h, solid-to-liquid ratio of 3:12.5 (g/mL)) in carrots with 2-MeTHF resulted in a 12% higher total carotenoid content compared to that obtained with *n*-hexane [70]. As already commented, 2-MeTHF has also been proven to be efficient for the extraction of carotenoids from wild-type and phytoene-accumulating *Chlorella sorokiniana* [25]. Overall, these studies suggest that 2-MeTHF could serve as a promising alternative to conventional organic solvents for the extraction of carotenoids from natural sources. 

### 5.3. Natural Deep Eutectic Solvents and Deep Eutectic Solvents

There are several definitions for describing natural deep eutectic solvents (NaDESs, or commonly named as NADES) and deep eutectic solvents (DESs), the most general being a mixture of two or more compounds associating through hydrogen bonding. The specific interactions among these constituents (the formation of a hydrogen bonding network, which increases the system’s stability and, in the case of ionic constituents, allows the charge delocalization) generally enable a significantly lower melting point of these solvents compared to those of their constituents. Thus, the melting points of some NaDESs (mainly those containing amides, carboxylic acids, and sugar-derived polyols with organic salts) are even below room temperature.

Some factors also result in decreases in the melting points of these solvents. The asymmetry of the cationic constituents lowers the melting point due to lower lattice energy, while increasing the electron affinity of the anionic constituents also lowers the melting point as it results in stronger hydrogen bonds between the two components. Furthermore, the addition of a ternary component to the system or stronger interactions between the components can also lead to a decrease in the melting point. It has been observed that NaDES components with lower molecular weight have a greater melting point depression [88]. NaDESs are a type of DES that are synthesized from natural components, such as sugars, making them more environmentally friendly than DESs [89]. Both solvents have unique properties, such as their polarity, ionic conductivity, and phase behaviour, that make them promising alternatives to traditional solvents in various extraction and processing applications. NaDESs are characterized by a high viscosity, which can be a limitation for industrial applications, although this can be addressed by adjusting the water content or solvent temperature. DESs, on the other hand, are characterized by low volatility and non-flammability, which make them ideal for lignocellulosic biorefinery applications [90,91].

DESs are classified into four different types (type I–IV) depending on the components used in their synthesis, water miscibility, and pH conditions. However, a new type V DES has recently been proposed. These V-type DESs can be easily recovered and revitalized through an evaporation process. This attribute makes them a more practical option for use in certain industrial bioprocesses, unlike other DES varieties [90]. 

DESs and NaDESs have emerged as promising alternatives to conventional solvents for the extraction of natural compounds, such as carotenoids [92]. To date, there are 17 and 22 published scientific articles in which carotenoids were extracted with NaDESs and DESs, respectively (Figure 2). Recently, DESs have been tested for the extraction of β-carotene from millet via grinding-assisted extraction (40 s, 1:4 g/mL of solid–liquid ratio, 2,6-di-*tert*-butyl-4-methylphenol-assisted (3 mg/mL)), using *N,N*-dimethylcyclohexylamine (DMCHA-B), *N,N*-dimethyloctylamine (DMOA-B), and *N,N*-dimethylbenzylamine (DMBA-B) as hydrogen bond acceptors and *n*-butanol as a hydrogen bond donor, in a 3:1 proportion. The study reported that the contents of β-carotene extracted using these DMCHA-B, DMOA-B, and DMBA-B were 2.7, 3.1, and 3.3 times higher than that obtained with ethanol, respectively [73]. Similarly, in a study with tomato powder, lycopene extraction was carried out via conventional extraction (maceration with magnetic stirring, 62 min, a solvent-to-solid ratio of 64:1 (*v*/*w*)) using a NaDES based on terpenes and fatty acid mixture (capric acid/lauric acid, 1:2). The study stated that the carotenoid extraction efficiency of the NaDES was either equal to or higher than that of acetone (up to 1.08 times greater) [74]. Finally, in a recent study performed by Viñas-Ospino et al. (2023), the UAEs (120 W ultrasound intensity, 1:20 (*w*/*v*) solid–solvent ratio, 20 min) of carotenoids from orange peel using several NaDESs (choline chloride/urea, proline/malic acid, *l*-menthol/*d,l*-camphor, *l*-menthol/eucalyptol, and lauric acid/octanoid acid) were compared. Among these NaDESs, menthol/camphor (1:1) was the most promising solvent regarding its stability and yield [19]. Overall, these studies highlight the potential of DESs and NaDESs as a viable alternatives to conventional solvents for the extraction of carotenoids.

### 5.4. Ionic Liquids

Ionic liquids (ILs), also known as Newton’s liquids, are composed of an organic cation and an organic or inorganic anion with a melting point below 100 °C, which are normally liquid at room temperature [93]. 

Due to their low vapour pressure, ILs have a low probability of releasing toxic substances into the atmosphere; nonetheless, ILs can still contaminate soil and groundwater, and the degree of adsorption depends on their lipophilicity/hydrophilicity and the length of the cationic alkyl chain. Their chemical and thermal stability make them highly resistant to decomposition, resulting in long-term persistence in the environment. In any case, the biodegradability of ILs varies depending on their constituent cations and anions. Water-soluble ILs, such as cholinium or carboxylates, facilitate biodegradation as they can dissolve in water and interact more easily with microorganisms. However, lipophilic ILs aid in transports across cell membranes of microorganisms, which may make them more persistent in the environment. Thus, the components of ILs should be carefully selected, as their properties can be adjusted to improve their persistence and degradability [94]. In summary, although some properties of ILs make them attractive from the point of view of green chemistry, there are currently doubts about whether they could be considered eco-friendly solvents [93].

In recent years, ILs have emerged as promising solvents for the extraction of carotenoids from various sources, including microalgae, plants, and food waste, resulting, in some cases, in higher yields and selectivity compared to conventional solvents, such as acetone or methanol [95]. Research on the extraction of carotenoids with ILs represents 28% of the articles published on the extraction of carotenoids with the solvents evaluated in this review (Figure 2). This percentage is higher than those published on NaDESs (20%) and DESs (26%) (Figure 2).

In a recent study, the recovery of β-carotene and astaxanthin from *Phaffia rhodozyma* (wet biomass) via solid–liquid extraction (0.2:1 (*w*/*v*) solid-to-liquid ratio, 60 min, 65 °C, stirring speed of 300 rpm) using a cholinium-based IL resulted in 1.9 and 1.7 times higher yields than that with acetone, respectively. However, this study suggested that concentrated ILs pose a challenge due to their high viscosity and the requirement for further techno-economic assessment before industrial implementation [75]. In another study, the extraction of lutein from *C. sorokiniana* via conventional extraction (maceration, 45 min, 20 °C) was effective using an alkyl carbamate IL (synthesized by mixing dipropylammonium dipropylcarbamate (DPCARB) and liquefied CO_2_, at a molar ratio of 2:1). An approximately three-fold higher lutein yield was observed with this CO_2_-based IL (DPCARB) compared to that of acetone, and an approximately 1.2 times higher yield was observed with DPCARB compared to that of methanol [76]. Moreover, in another study, tetrabutyl phosphonium hydroxide was used for the extraction of lutein from *Chlorella saccharophila* (40% (*w*/*v*) of ionic liquid concentration, 5 min extraction time, 0.5 mL/mg of solvent/biomass ratio, and 25 °C temperature), and the extraction yield was approximately 20.5 times higher compared to that of methanol [77]. In addition, the carotenoid extraction from orange peel via UAE (200 W, 20 kHz, 80% amplitude, 5 min, 1:3 solid/liquid ratio) was compared using acetone and different ILs (1-butyl-3-methylimidazolium chloride ([BMIM][Cl]), 1-n-butyl-3-methylimidazolium tetrafluoroborate ([BMIM][BF4]), and 1-hexyl-3-methylimidazolium chloride ([HMIM][Cl])). The total carotenoid contents of the extracts obtained with [BMIM][Cl], [BMIM][BF4], and [HMIM][Cl] were 2.3, 2.9, and 1.5 times higher than that obtained with acetone, respectively [96]. Although ILs offer potential as efficient and sustainable extraction solvents for natural carotenoids, addressing the associated challenges and optimizing extraction conditions for different carotenoid sources through further research is crucial. This will enable the production of high-value natural carotenoid products with applications in various industries [95].

## 6. Conclusions

Carotenoids are extracted with different organic solvents, including hexane, methanol, and DMSO, which can pose harm to health and the environment depending on their use and exposure. On the other hand, many conventional techniques for carotenoid extraction involve a high amount of solvent and energy consumption. Thus, there is increasing interest in improving the extraction of carotenoids to make it more sustainable. Different techniques (such as ultrasound, microwaves, enzyme treatment, pulsed electric fields, pressurized liquid extraction, and sub- and supercritical fluid extraction) are being tested, as well as innovative solvents including ionic liquids, deep eutectic solvents (i.e., either natural or not), and other green solvents deriving from renewable feedstocks (2-methyltetrahydrofuran, ethyl lactate). Among these methodologies, ultrasound-assisted extraction and extraction with supercritical fluids are those that have generated the greatest number of original scientific publications in relation to the extraction of carotenoids. In any case, there are still few studies that test these bio-based solvents for carotenoid extraction. 

In any case, much research is still needed, as well as technological, economic, and environmental assessments. Some aspects that require more attention are the purity and the composition of the extracts. Being highly lipophilic, carotenoids are extracted together with other lipophilic compounds. In addition, in general, there is not much selectivity in the type of carotenoid extracted when the source contains several of them. The extraction of several carotenoids and lipophilic compounds could be desirable or not depending on the research objective or industrial application. For instance, the fact that a carotenoid-rich extract exhibits certain properties or actions does not necessarily mean that they are attributable to them. On the other hand, there are occasions in which the extraction of a certain carotenoid or type of carotenoid is sought but cannot be achieved easily. One example would be the extraction of colourless carotenoids from sources in which they occur together with other coloured carotenoids. Maybe the customizable designs of NaDESs and DESs can offer advantages in these directions in the near future. In any case, although laboratory results are in many cases very positive, their scalability to real industrial scenarios remains, in many cases, an important obstacle, mainly due to techno-economic reasons (for instance, an insufficient production capacity, the cost of infrastructure, the cost of the final product, etc.). On the other hand, it is important to include life cycle assessment analyses to better assess whether innovative extraction processes are more sustainable than the processes that are applied in the industry.

## Figures and Tables

**Table 1:** Extraction mechanisms of the green extraction technologies.

Extraction Method	Mechanism	Ref
UAE	Utilizes sound waves for inducing cavitation in the solution, thereby facilitating the extraction process.	[11]
MAE	Generates heat within the solvent by employing ionic conduction of the dipole rotation and dissolved ions in the polar solvent.	[12]
PEF	Applies high-voltage microsecond pulses to induce pores in cell membranes, resulting in the disruption of barrier function and leakage of intracellular content.	[13]
PLE	Utilizes organic solvents under high pressures and temperatures, exceeding the boiling point, amplifying the solubility of analytes, and reducing solvent viscosity. This approach minimizes the required time and solvent volume.	[14]
SuperFE	Utilizes CO_2_ in supercritical state as extraction solvent, segregating the analytes according to their relative solubility. Supercritical CO_2_ has a high density and solvent power, similar to that of a liquid.	[15]
SubFE	Extracts less-polar compounds using water or other fluids in subcritical conditions under high pressures and high temperatures, sustaining subcritical fluids in a liquid state for a brief extraction period.	[16]
EAE	Encompasses the binding of cells to the active site of an enzyme, which induces a transformation of the enzyme form to adapt to the substrate. Consequently, the active components are released from the cells into the extraction medium.	[17]

**Table 2:** Main results of studies on carotenoid recovery with green extraction methodologies.

Extraction Method	Conditions	Solvent	Matrix	Results	Ref
CSE and UAE (bath)	CSE (290 rpm) and UAE (40 kHz, 80 W): time (6, 10, 20, 30, 34 min), SSR (6 mL/59, 80, 130, 180, 201 mg).	Acetone, ethanol, petroleum ether, and methanol	Cashew apple	Optimal conditions: CSE: 38% acetone, 30% ethanol, and 32% petroleum ether, 23 min, and 136 mg; UAE: 44% acetone and 56% methanol, 19 min, and 153 mg. UAE achieved a ~21% faster and higher carotenoid yield in all samples compared to CSE.	[18]
CSE, MAE, and UAE (probe)	CSE: 30 min, RT, SSR: 10 mL/g, 150 rpm; MAE: 100 W, 30 min, 60 °C, SSR: 1 g/10 mL. UAE: 40 kHz, 50 W, 60%, 30 min, SSR: 10 mL/g; UAE + MAE (same conditions).	Menthol/camphor (1:1, n:n)	Orange peel	Significantly higher extraction with UAE (~2-fold) compared to CE, no statistical differences between CE and MAE or between UAE and UAE+MAE.	[19]
UAE (probe)	20 kHz, 500 W, amplitude (20, 30, 40, 50, 60, 70%), time (10, 15, 20, 25, 30, 35 min), temperature (55, 60, 65, 70, 75, 80 ℃), SSR (10, 20, 30, 40, 50, 60 mL/g).	Ethyl lactate, limonene, soybean oil, and sunflower oil	*Sargassum fusiforme*	Optimal conditions: ethyl lactate, 20 kHz, 500 W, 53%, 27 min, 75 ℃, and SSR: 40 mL/g.	[20]
CSE and UAE (bath)	CSE: 90 min, 40 °C, SSR: 40 mL/g; UAE: 45 kHz, ultrasonic power (150, 180, 210 W), time (30, 40, 50 min), and SSR (30, 35, 40 mL/g).	Ethanol–petroleum ether mixture (2:1, *v*/*v*)	*Cucurbita moschata*	Optimal UAE conditions: 45 kHz, 203 W, 30 min, and 31 mL/g. Compared to the CSE, UAE avoided degradation and isomerization, resulting in a higher yield.	[21]
UAE (probe)	20 kHz, amplitude (20, 40, 60, 80, 100%), time (10, 20, 30, 40 min), SSR (30, 40, 50, 60 mL/g), and hexane/acetone ratio (50/50, 70/30, 90/10).	Hexane/acetone mixture	Cantaloupe waste	Optimal UAE conditions: 20 kHz, 100%, 10 min, 55 mL/g, and hexane/acetone ratio of 80/20.	[22]
UAE (bath)	40 kHz, 80 W, time (14, 20, 30, 40, 44 min), and SSR (10 mL/10, 30, 80, 130, 150 mg).	Acetone/ethanol (3:1)	Buriti (*Mauritia flexuosa*)	Optimal UAE conditions: 40 kHz, 80 W, 30 min, 80 mg/10 mL, acetone/ethanol (75:25).	[23]
UAE (probe)	20 kHz, 70% amplitude, time (3, 10, 20, 30, 37 min), temperature (10, 20, 35, 50, 60 °C), SSR (30, 50, 70 mL/g), solvent percentage (13, 30, 55, 80, 97%).	Ethanol, methanol, acetone, acetonitirile, and *n*-hexane	Carrot pomace	Optimal UAE conditions for total carotenoid content: 17 min, 32 °C, 50 mL/g, and 51% ethanol.	[24]
UAE (probe)	UAE: 20 kHz, 30%, 2 min, SSR: 2 mL/0.1 g.	Ethanol, methanol, ethyl lactate, MeTHF, and DMSO	*Chlorella sorokiniana*	The best solvent depended on the matrix (fresh: ethanol; freeze-dried: methanol; encapsulated: MeTHF and ethyl lactate).	[25]
CSE and MAE	CSE: 30 min, 60 °C, 100 mg/5 mL (10 M aqueous KOH with 2.5% ascorbic acid); MAE: time (0, 15, 30, 45, 60, 180, 300, 600 s), 60 °C, SSR (1 mL/10, 20, 30, 40, 50, 60 mg), KOH concentration (0, 2, 4, 6, 8, 10, 12 M).	Ethanol	*Chlorella sorokiniana*	Optimal alkali-assisted MAE pretreatment conditions for lutein: 850 W, 1.47 min, 8.16 M KOH, and SSR of 36.8:1 mg/mL. Lutein yield obtained via MAE was 3.26 folds higher than that obtained via CSE.	[26]
MAE	Power (50, 80, 125, 170, 200 W), time (1, 3.14, 6.3, 9.46, 12 min), and oil-to-waste ratio (5:1, 8:1, 12.5:1, 17:1, 20:1 g/g).	Flaxseed oil	Carrot juice	Optimal conditions: 165 W, 9.39 min, and SSR 8.06:1 g/g.	[27]
MAE and UAE (probe)	MAE: power (30, 40, 50 W), time (5, 7, and 9 min), and SSR (10, 20, 30 mL/g); UAE: 20 kHz, power (375, 562.5, 750 W), time (2, 9, 16 min), and SSR (10, 30, 50 mL/g).	MAE: acetone, ethanol, petroleum, ether/acetone/ethanol mixture (2:1:1), and *n*-hexane/acetone/ethanol mixture (2:1:1); UAE: acetone, *N,N*-dimethylformamide, isopropanol/*n*-hexane mixture (1:1), petroleum ether/acetone mixture (1:1), and petroleum ether/acetone/ethanol mixture (2:1:1)	*Aristeus antennatus* shrimp	Optimal MAE conditions: 30 W, 7 min, 20:1 mL/g, and *n*-hexane/acetone/ethanol 2:1:1 (*v*/*v*/*v*); Optimal UAE conditions: 20 kHz, 600 W, 5 min, 10:1 mL/g, and acetone. No differences between UAE and MAE in carotenoid extraction from the head of the shrimp. UAE resulted in a ~2 times higher total carotenoid extraction compared to that of MAE in the body of the shrimp.	[28]
PEF	3.5 kW of power (15 kV/cm, 150 μs).	Ethanol	*Rhodotorula glutinis*	PEF treatment without incubation did not recover carotenoids; however, PEF with 1 h incubation (20 °C) permits the extraction of carotenoids from fresh biomass.	[29]
Bead beating, freeze-thawing, thermal treatment, PEF, and UAE (probe)	PEF: 1 Hz, 1 kV/cm, 10 pulses, 50 kJ/Kg; Bead beating: 4800 rpm (5–10 cycles of 60 s), 6 h incubation time; UAE: 450 W, 80%, 10 times for 10 s; Thermal treatment: 1 h, 70 °C; Freeze-thawing: in liquid nitrogen and left to melt on ice (repeated 5 times).	Ethanol, acetone, and methanol	*Haematococcus pluvialis* and *Chlorella vulgaris*	The best extraction yields were achieved after PEF pretreatment with 6 h incubation and ethanol. Statistically significant differences in the extracted carotenoid yields after PEF pretreatment compared to other treatments in *H. pluvialis* cells grown in the control BBM medium or in N-free BBM medium supplemented with 6 g/L glucose.	[30]
CSE and PEF	CSE: 25 °C, 120 rpm, SSR: 100 mL/5 g, hexane/ethanol/acetone (50:25:25); PEF: 1 Hz, 5 kV/cm, 90 µs, time (20, 160, 300 min), SSR: 5 g/100 mL, hexane/ethanol (25:75, 50:50, 75:25).	Acetone, hexane, and ethanol mixtures	Tomato waste	Optimal PEF conditions: 1 Hz, 5 kV/cm, 90 µs, 150 min, and 30% hexane. PEF treatment improved the carotenoid extraction by 39% as compared with CSE in a mixture of hexane/ethanol/acetone (50:25:25).	[31]
CSE and PEF	CSE: 100 µL sample/1 mL solvent, vortexed; PEF: 0.5 Hz, 20 kV/cm, 75 µs, with or without 1 h incubation (20 °C).	Ethanol (96%)	*Chlorella vulgaris*	Extraction yield with PEF was significantly higher than that with CSE. Pre-incubation for 1 h after PEF treatment improved extraction yields.	[32]
ASE, CSE, PEF, and PEF+ASE	CSE: 400 rpm, 30 min, RT, SSR (1:10, *w*/*v*); PEF: 100 kJ/kg, 74 pulses, 300 mL of tap water/30 g; PEF+ASE: 1 min of preheating period, 5 min of heating period, 60% of flush volume, 10 MPa of extraction pressure (for 15 min) and 60 s of nitrogen purge, 50 °C.	DMSO and ethanol	Shrimp by-products	PEF+ASE with DMSO resulted in the highest carotenoid recovery from *Aristeus antennatus*. The antioxidant capacity varied depending on solvent.	[33]
CSE and PLE	CSE: 15 min, 40 °C, SSR (20 mL/0.5 g); PLE: preheating time: 1 min, heating time: 5 min, 40 °C, SSR (0.5 g/20 mL).	DMSO (0, 30, 50, and 100%)	*Spirulina, Chlorella* and *Phaeodactylum tricornutum* powder	Carotenoid recovery with PLE was significantly higher than that with CSE; 100% DMSO enhanced the extraction yields of carotenoids significantly.	[34]
CSE, PEF, PLE, and PEF+PLE	CSE: 6 h, 40 °C, SSR: 20 mL/g (chloroform/methanol (5:2, *v*/*v*)); PEF: 3 kV/cm, 44 pulses, 99 kJ/kg energy input, SSR: 200 mL H_2_O/2 g; PLE: 15 min, 40 °C, 1 min preheating period, 5 min heating period, flush volume of 60%, nitrogen purge of 60 s, 103.4 bars,1000–2000 μS/cm, SSR: 20 mL/0.5 g (H_2_O, 50% DMSO, 100% DMSO).	H_2_O, 50% DMSO, 100% DMSO	*Spirulina* biomass	The extraction yield with PEF + PLE (50% DMSO) was significantly higher than those with PEF, PLE, or CSE. PEF + PLE increased efficiency and reduced the extraction time.	[35]
PLE	20 min, temperature (50, 100, 150 °C), 103.4 bar, nitrogen purge 300 s.	Ethanol, ethyl acetate, and *n*-hexane	*Chlorella vulgaris* and *Phaeodactylum tricornutum* (wet and dry biomasses)	Best conditions for total lipid yield: ethyl acetate and *n*-hexane (temperature did not affect) for dry *C. vulagris*; ethyl lactate at 150 °C for wet *C. vulgaris;* ethanol at 100 or 150 °C for dry *P. tricornutum*; and ethanol at 150 °C for wet *P. tricornutum.*	[36]
CSE and SuperFE	CSE: 30 min, 40 °C, 1 mL/g; SuperFE: 40−60 °C, CO_2_ flow rate 16.5 g/min, equilibration time (0−30 min), extraction pressure (200−500 bar), and ethanol content in supercritical fluid mixture (8−13 wt %), with or without MW pretreatment (time (30–90 s), temperature (41–140 °C), up to 300 W).	Acetone	Brown crab (*Cancer pagurus*) processing waste	Optimal SuperFE conditions: 500 bar, 40 °C, and 13 wt % ethanol content after an optimized MW pretreatment (140 °C, 90 s, 300 W).	[37]
CSE, MAE, and SuperFE	CSE: 24 h, 30 °C, 500 rpm, SSR: 37 mL/g; MAE: time (5–25 min), temperature (40–60 °C), power (300–800 W), SSR (20–90 mL/g); SuperFE: 60 °C, 250 bar, flow rate 40 g/min, total solvent consumption 100 kg CO_2_/kg biomass.	Ethanol 90% (*v*/*v*)	*Chlorella vulgaris*	Optimal MAE conditions: 14 min, 60 °C, 300 W, and 22 mL/g. CSE presented the highest yield. MAE and SFE led to an antioxidant capacity similar to or better than CSE in a significantly shorter extraction time.	[38]
SuperFE	Extraction time (60, 90, 135, 180, 210 min), temperature (55, 60, 70, 80, 85 °C), pressure (250, 300, 375, 450, 500 bar), SSR 1 mL/0.1 g, flow rate CO_2_ (35 g/min), and rice bran oil (3%, *w*/*w*).	CO_2_ with rice bran oil as co-solvent	*Citrus paradise Macfad*	Optimal conditions for lycopene extraction: 143 min, 64 °C, and 325 bar. Extraction temperatures higher than 80 °C and time lower than 180 min led to lycopene isomerization.	[39]
SuperFE	80 min, temperature (50, 60, 70 °C), pressure (150, 250, 350 bar), CO_2_ flow rate 15 g/min, and co-solvent concentration (5, 10, 15%, *v*/*v*).	CO_2_ with ethanol as co-solvent	Carrot peels	Optimal conditions: 59 °C, 349 bar, and 15.5% ethanol.	[40]
SubFE	15 min, temperature (303, 318, 333 K), pressure (5, 11, 17 MPa), co-solvent percentage (2, 4, 6%), 50 min with a constant flow rate of 10 g/min.	1,1,1,2-Tetrafluoroethane with ethanol as co-solvent	*Laminaria japonica*	Optimal conditions: 324.13 K, 17 Mpa, and a co-solvent amount of 4.73%.	[41]
SubFE and UAE (bath)	UAE (to select best solvent): 15 min, 35 °C, SSR: 10 mL/0.5 g. SubFE: extraction time (30, 60, 90 min), extraction temperature (35, 50, 75 °C), pressure of 20 MPa, SSR (200:1, 100:1, 20:1 (v/wet weight).	Hexane, DCM, ethanol, methanol, THF, ultrapure water, and THF:DCM (1:1).	*Phaeodactylum tricornutum*	Optimal conditions: methanol, 60 min, 35 °C, 20 MPa, 120 rpm, and SSR 200:1.	[42]
SOX and SubFE	SOX: 360 min, temperature (341.15 K for *n*-hexane, and 333.15 K for petroleum ether), SSR: 200 mL/ 5 g, solvent removed at 316.15 K; SubFE: 60 min, temperature (293.15, 313.15, 333.15 K), pressure (2, 6, 10 MPa), propane flow rate of ~2 cm^3^/min.	SOX: *n*-hexane or petroleum ether; SubFE: propane	(*Maximiliana maripa*) pulp oil	Optimal SubFE conditions: 293.15 K and 2 MPa. Extraction yield with SOX (*n*-hexane) was significantly higher than that with SubFE.	[43]
SOX, SubFE, and SuperFE	SuperFE: 52.5 °C, 27.50 MPa, 5 mL CO_2_/min flow rate; SubFE: 12 h, 29 °C, 6.8 MPa, 250 extraction cycles; SOX: 12 h, 70 °C.	SuperFE: CO_2_; SubFE: ethanol; SOX: *n*-hexane	Saudi date fruit flesh (Sukari, Ambara, Majdool, and Sagai date fruit)	The highest carotenoid extraction was found as follows: Sukari (via SubFE) and Ambara, Majdool, and Sagai date fruit (via SuperFE).	[44]
EAE	Pretreatment time (0.5, 2, 3.5, 5, 6.5 h), extraction time (0.5, 1.5, 2.5, 3.5, 4.5 h), extraction temperature (10, 20, 30, 40, 50 °C), enzyme solution-to-solid ratio (10, 20, 30, 40, 50 dm^3^/kg), and enzyme load (0, 0.05, 0.1, 0.15, 0.2 kg/kg); SSR: 30 mL/0.5 g.	Hexane, enzyme/Peclyve PR and Cellulyve 50LC (50:50)	Tomato waste	Optimal conditions: 3.18 h, 30 °C, and 0.16 kg/kg enzyme load.	[45]
EAE	Time (12, 18, 24 h), temperature (30, 33.5, 37 °C), enzyme dose (0.15, 0.3, 0.45, 0.6, 0.75 mL), pH (4.6, 6, 7.4).	95% Ethanol, enzyme/fructozym^®^ MA	Carrot	Optimal conditions: 24 h, 37 °C, pH 7.4, and 0.3 mL enzyme dose.	[46]
CSE and EAE	CSE (to select the best solvent): 30 min, RT, SSR: 20 mL/4 g; EAE: time (1, 2.5, 4 min), temperature (40, 50, 60 °C), SSR (1 mL/5, 17.5, 30 g), enzymatic reaction time (1, 3, 5 h), enzyme/substrate ratio (0.2, 1.1, 2 mL/g), enzyme/enzyme ratio (1, 2, 3).	CSE: acetone, ethyl acetate, ethanol, and 1:1 combinations, and ethanol/water mixture (1:1); EAE: ethyl acetate, enzymes: pectinolytic enzyme (P), cellulolytic enzyme (C), and a combination of carbohydrases, including arabanase, cellulase, β-glucanase, hemicellulase, and xylanase (V)	Tomato waste	Optimal EAE conditions: C-P combination for enzymatic pretreatment, ethyl acetate as solvent, 1 h extraction time, 40 °C, 5 h enzymatic reaction time, 0.2 mL/g enzyme/substrate ratio, 5 mL/g solvent/substrate ratio, and 1 enzyme/enzyme ratio.	[47]

ASE: accelerated solvent extraction; BBM: Bold’s basal medium; CH: Conventional heating; CSE: Conventional solvent extraction; DCM: dichloromethane; DMSO: dimethylsulfoxide; EAE: enzyme-assisted extraction; MAE: microwave-assisted extraction; MeTHF: 2-methyltetrahydrofuran; MW: microwave; PEF: pulsed electric field; PLE: pressurized liquid extraction; RT: room temperature; SOX: Soxhlet extraction; SSR: solvent-to-solid ratio; SubFE: subcritical fluid extraction; SuperFE: supercritical fluid extraction; THF: tetrahydrofuran; UAE: ultrasound-assisted extraction.

**Table 3:** Main results of studies on carotenoid extraction with green solvents.

Solvent	Extraction Method	Conditions	Matrix	Results	Ref
Ethyl lactate, acetone, ethyl acetate, hexane, and ethanol	CSE	SSR: 10 mL/g, extract removed at different time intervals (5–40 min), and temperatures (25, 50, 70 °C).	Tomato waste	Optimal conditions: ethyl lactate, 30 min, and 70 °C.	[65]
Ethyl lactate/ethanol (3:2)	CSE	Time (10–40 min), temperature (10–30 °C), 360 rpm, and crude palm oil proportion (20–60%).	Palm olein	Optimal conditions: 10 min, 20 °C, and 50% of crude palm oil.	[66]
Hexane, ethyl acetate, ethyl lactate, and ethyl acetate/ethyl lactate (1:3, *v*/*v*).	UAE (bath)	10 min, 35 °C, and 1 g/20 mL.	Tomato by-products	The highest lycopene extraction was obtained with ethyl lactate in the wet sample.	[67]
Ethyl lactate and ethyl lactate/ethanol mixtures (0–100%)	CSE	Time (0–350 min), temperature (30, 45, and 60 °C), and SSR (0.25–1.0 g/10 mL).	Tangerine and red tomato, corn, and carrot	Optimal conditions: lutein (2 h, 30 °C), β-carotene (0.5 h, 30 °C), and lycopene (1 h, 45 °C). The addition of *R*-tocopherol or *R*-lipoic acid improves the extraction efficiency. Ethyl lactate is an excellent solvent for extracting *trans*- and *cis*-lycopene isomers from dried tomato powder.	[68]
Ethanol, acetone, ethyl lactate, sunflower oil, and water	UAE (bath) and heating	UAE: 35 kHz, 30 min, and 40 °C; Heating: 30 and 60 min and 45 and 100 °C.	Lycium fruits (Goji, naturally dried and freeze-dried)	The highest carotenoid levels were obtained via UAE (with water and ethyl lactate) and heating (with ethyl lactate). The lowest content was obtained with sunflower oil, ethanol, and acetone. Water was a better solvent for naturally dried berries and ethyl lactate and sunflower oil for the freeze-dried sample.	[69]
MeTHF, dimethyl carbonate, cyclopentyl methyl ether, isopropyl alcohol, ethyl acetate, and *n*-hexane	CSE	1 h maceration, 65 °C, and 30 g/125 mL.	*Daucus carota*	The best green solvents were as follows in descending order: cyclopentylmethyl ether, MeTHF, and ethyl acetate.	[70]
MeTHF, hexane, tetrahydrofuran, acetone, and 1,4- dioxane	CSE	First maceration overnight with SSR 1:10 (*w*/*v*) and second maceration with SSR 1:2 (*w*/*v*).	*Tagetes erecta L.*	MeTHF is a potential green alternative solvent to hexane and tetrahydrofuran for lutein extraction.	[71]
Hexane, dry MeTHF, and MeTHF 95.5%	SOX	4.5 h.	Olive pomace extracts	Dry MeTHF and MeTHF 95.5% recovered carotenoid yields ~11.3 and 12.4 folds higher, respectively, than that of hexane.	[72]
HBA:HBD (3:1), being HBA ethanol, *N,N*-dimethylcyclohexylamine, *N,N*-dimethyloctylamine, and *N,N-*dimethylbenzylamine and HBD *n*-butanol	GAE, WBE, and UAE	GAE: 30 Hz, 40 s, and SSR 1:4; WBE: 120 min, 50 °C, and SSR 1:4; UAE: 40 kHz, 15 min, 50 °C, and SSR 4 mL/g.	Millet	DESs extracted a significantly higher carotenoid yield compared to ethanol. GAE resulted in the best extraction method.	[73]
Acetone, DL-menthol or thymol, and capric acid as HBAs; capric acid and lauric acid as HBDs	CSE	60 min, RT, 750 rpm, and SSR 40 mL/g.	Tomato	The combination of capric acid and lauric acid exhibited extraction capacities comparable to that of acetone.	[74]
Acetone; cholinium-based ILs: choline bicarbonate (Ch), octanoic acid (Oct), hexanoic acid (Hex); butyric acid (But), and lactic acid (Lac)	CSE	Time (0, 20, 60, 120, and 144 min), temperature (11.4, 25, 45, 65, and 78.4 °C), enzyme concentration (0, 20, 50, 80, and 100 wt %), and SSR: 0.2 g/1 mL.	*Phaffia rhodozyma*	Optimal conditions: [Ch][Oct], 60 min, 45 °C, and 50 wt % in water.	[75]
Acetone, methanol, dipropylammonium dipropylcarbamate (DPCARB), diallylammonium diallylcarbamate (DACARB), and dibutylammonium dibutylcarbamate (DBCARB)	CSE	Time (30, 45, 60, 75, and 90 min), temperature (25, 35, 45, and 55 °C), and DPCARB/methanol ratio (9:1, 8:2, 7:3, 6:4).	*Chlorella sorokiniana*	Optimal conditions: DPCARB/methanol (9:1), 45 min, and 25 °C.	[76]
Methanol, IL1 [1-decyl 3 methyl imidazolium chloride], IL2 [tetrabutyl phosphonium hydroxide], IL3 [tetrabutyl hexadecyl phosphonium bromide], and IL4 [tetrabutyl ammonium hydroxide]	CSE	Time (5, 10, and 15 min), temperature (25, 40, and 55 °C), IL concentration (5, 22.5, and 40%), SSR (0.1, 0.3, and 0.5 mL/mg).	*Chlorella saccharophila*	Optimal conditions: IL2, 5 min, 25 °C, 40% IL concentration, and 0.5 mL/mg SSR.	[77]

CSE: Conventional solvent extraction; GAE: grinding-assisted extraction; HBA: hydrogen bond acceptor; HBD: hydrogen bond donor; ILs: Ionic liquids; MeTHF: 2-methyltetrahydrofuran; RT: room temperature; SOX: Soxhlet extraction; SSR: solvent-to-solid ratio; UAE: ultrasound-assisted extraction; WBE: water bath extraction.

**Table 4:** Physical and chemical properties of 2-methyltetrahydrofuran and ethyl lactate.

Solvent	2-Methyltetrahydrofuran	Ethyl Lactate
Molecular formula	C_5_H_10_O	C_5_H_10_O_3_
Molecular weight (g/mol)	86.132	118.131
Appearance	Colourless	Colourless
Stability	Stable, but highly flammable. Not suitable for use with oxidizing agents, potent acids, or strong bases. There is a risk of developing explosive peroxides during storage, which is why it is frequently supplied with an inhibitor as a precaution.	Stable, flammable, and not compatible with powerful oxidizing agents.
Toxicity	LD50 orally in Rabbit: >300–2000 mg/kg. LD50 dermal Rat > 2000 mg/kg	LD50 orally in Rat (female): >2.000 mg/kg. LD50 dermal Rabbit: >5.000 mg/kg
Safety	Flammable, corrosive, and irritant. Acute toxicity. It requires safety glasses, good ventilation, a test for the presence of peroxides before use, and the removal of ignition sources from the working area.	Flammable, corrosive, and irritant for eyes and skin.
Melting point (°C)	−136	−26
Boiling point (°C)	82	154
Organic solvent solubility	Miscible	Miscible
Water solubility	Immiscible	Miscible (with partial decomposition)
Density (g/mL)	0.855	1.03
Refraction index	1.407	1.415
Dielectric constant	6.97	13.1
Molar refraction (mL/mol)	24.8	28.72
Dipole moment (D)	1.38	2.55

LD50, median lethal dose. Data obtained from ChemSpider, ChemicalBook, and Stenutz [81,82,83].
